# The Double-Edged Sword of Urbanization and Its Nexus with Eco-Efficiency in China

**DOI:** 10.3390/ijerph17020446

**Published:** 2020-01-09

**Authors:** Li Yue, Dan Xue, Muhammad Umar Draz, Fayyaz Ahmad, Jiaojiao Li, Farrukh Shahzad, Shahid Ali

**Affiliations:** 1 School of Economics, Lanzhou University, Lanzhou 730000, China; mgliang@lzu.edu.cn (L.Y.); xuedan2748409@163.com (D.X.); lijj2017@lzu.edu.cn (J.L.); 2 Department of Management and Humanities, Universiti Teknologi PETRONAS, Seri Iskandar, Perak 32610, Malaysia; umardraz2626@gmail.com; 3 School of Economics and Management, Guangdong University of Petrochemical Technology, Maoming 525000, China; Farrukh.hailian@gmail.com; 4 School of Management, Xi’an Jiaotong University, Xi’an 710049, China; shahidali24@hotmail.com

**Keywords:** China, eco-efficiency, urbanization, Super-SBM, spatial econometric

## Abstract

Urbanization has made tremendous contributions to China’s economic development since its economic reforms and opening up. At the same time, population agglomeration has aggravated environmental pollution and posed serious challenges to China’s environment. This article empirically investigates the impacts of China’s urbanization on eco-efficiency, comprehensively reflecting economic growth, resource input, and waste discharge. We first measured the provincial eco-efficiency in China from 2005 to 2015 using the Super Slack-Based model (Super-SBM). We then constructed a spatial model to empirically analyze the effects of urbanization on eco-efficiency at the national level, and at four regional levels. The results indicated that the regional eco-efficiency in China has fluctuated, but is generally improving, and that a gap between regions was evident, with a trend toward further gap expansion. We observed an effect of spatial spillover in eco-efficiency, which was significant and positive for the whole country, except for the western region. The influence of urbanization on China’s eco-efficiency exhibited a U-curve relationship. The changing trend in the eastern, central, and western regions was the same as that in the whole country; however, the trend exhibited an inverted U-curve relationship in the northeastern region. To the best of our knowledge, covering a time period of 2005–2015, this article is the first of its kind to study the impact of urbanization on eco-efficiency in China at both the national and regional levels. This study may help policy-makers to create sustainable policies that could be helpful in balancing urbanization and the ecological environment.

## 1. Introduction

Since the reform and opening up 40 years ago, China’s economic and social development has made amazing progress, and China’s urbanization has also achieved rapid development [1]. The level of urbanization has improved from 17.90% (1978) to 59.58% (2018), and urbanization has made tremendous contributions to China’s economic development [2]. However, urbanization poses challenges to China’s ecological environment [3]. First, population agglomeration has increased waste production [4]. Second, due to the characteristics of the economy of Chinese provinces, some local governments are more inclined to pursue economic development and efficiency at the cost of the ecological environment. Cities are industrial layout centers, and exhibit a greater density of waste emissions and resource inputs than rural areas.

Although China is increasingly more attentive to environmental protection and pollution prevention, environmental quality has not significantly improved [5]. At present, China is in the mid-term of urbanization, and industrial enterprises with high pollution and energy consumption still play a significant role in economic development [6]. Chinese president Xi has pointed out in the Nineteenth National Congress of the Communist Party of China (CPC) that China’s green mountains and clear rivers are mountains of silver and gold, and that we should strive for the harmonious coexistence of humans and nature, and treat the environment as we treat our lives. Indeed, China’s economic growth is making a transition from pursuing “high-speed only” to paying more attention to economic growth quality [7]. At this stage, the potential downward pressure on economic growth has increased, while the pressure on resources and the environment has also increased. China urgently needs to protect its ecological environment, while promoting high-quality economic development.

Since Schaltegger and Sturm first proposed the concept of eco-efficiency in 1990 [8], many scholars and institutions have conducted in-depth research on eco-efficiency [9,10,11]. The definition of the World Business Council for Sustainable Development (WBCSD) has been widely accepted. The WBCSD described eco-efficiency as the provision of products and services, with price competitiveness, that meet human needs and improve quality of life, while at the same time the ecological impact and resource intensity of the entire life cycle are gradually reduced to a level consistent with the estimated carrying capacity of the Earth, to eventually achieve the goal of coordinated development of the environment and society [12]. The relationship between urbanization and eco-efficiency is complicated and varied [13]. On the one hand, with the development of urbanization, air pollution, water pollution, noise pollution, traffic jams, housing congestion, and other environmental problems have become increasingly prominent, which will pose challenges to the eco-environment. On the other hand, urbanization can improve the per capita income, education level, and people’s happiness, which will promote the improvement of eco-efficiency.

In this article, we first measure the provincial eco-efficiency in China from 2005 to 2015 using the Super Slack-Based model (Super-SBM); we then explore the spatial correlations and spatio-temporal changes in eco-efficiency. Finally, we expand the classical Stochastic Impacts by Regression on Population, Affluence, and Technology (STIRPAT) model and construct a spatial durbin model to empirically evaluate the effect of urbanization on provincial eco-efficiency in China. We expand the existing research to explore the mechanisms behind the effects of urbanization on eco-efficiency in China, using provincial data and from the perspective of spatial econometrics. The results will be useful to China’s provincial local governments, as this study comprehensively considers economic growth, resource input, and waste discharge, while measuring eco-efficiency. Likewise, it describes eco-efficiency in the process of urbanization more comprehensively and objectively. Finally, the results and conclusions will be critical to efforts addressing the reality of low per capita resources and poor environmental quality in China at the provincial level.

The remainder of this paper is divided into the following parts: Section 2 comprises the review of existing studies; Section 3 deals with the data and methodology; Section 4 offers statistical results and discussion; and Section 5 presents the conclusions and policy implications.

## 2. Literature Review

### 2.1. The Measurement of Eco-Efficiency

Studies evaluating eco-efficiency have attracted much attention. Methods for measuring eco-efficiency include the indexes system method [14], stochastic frontier analysis (SFA) [15], life-cycle assessment [16], and data envelopment analysis (DEA). Among the above methods, the DEA method is the most common method. Kuosmanen and Kortelainen [17] applied the DEA model to measure the eco-efficiency of road transportation in eastern Finland’s three largest towns. Korhonen and Luptacik [18] assessed the eco-efficiency of European power plants using DEA models. Huang et al. [19] used the DEA model to measure the eco-efficiency of 273 cities in China. Zhang et al. [20] used the three-stage DEA to measure the industrial eco-efficiency of 30 provinces in China from 2005 to 2013. Bai et al. [21] applied the super-efficiency data envelopment analysis (SEDEA) model to calculate the urban eco-efficiency of 281 prefecture-level cities in China from 2006 to 2013.

However, the traditional DEA models are mostly radial or angular. They do not consider the slack problem of input or output, and cannot achieve a true and effective measurement of the efficiency value when an undesirable output is included. Tone [22] proposed a non-radial, non-angled slacks-based measure (SBM) DEA model, which not only considered the improvement between the current state and the strong target value of the invalid decision-making units (DMUs), but also considered the slack improvement, avoiding deviations and effects caused by radial and angular models. Additionally, the SBM model also can measure efficiency when the model includes undesirable outputs. Tone [23] also proposed a Super-SBM model, which solves the difficulty in ranking the DMUs and allows the effective DMUs to have an efficiency value greater than 1, avoiding the problem of the effective DMUs being unable to be compared. Zhou et al. [24] estimated the eco-efficiency in Guangdong province based on Super-SBM. In this paper, we apply the Super-SBM model, which takes into account undesirable outputs, to evaluate the provincial eco-efficiency in China.

### 2.2. The Process of Urbanization

Urbanization can be defined as the expansion process of the urban population and urban scale, as well as a series of corresponding economic and social changes [25]. Since the reform and opening up, China’s urbanization has accelerated rapidly. The urban population increased from 170 million in 1978 to 831 million in 2018. As the largest developing country in the world, China’s urbanization has attracted the attention of many scholars [26,27]. Guan et al. [28] believe that China’s urbanization process is unique, and perhaps the greatest human habitation experiment in the world history, changing China’s society in an unprecedented way and in a short time. Indeed, rapid urbanization has played an important role in creating employment opportunities, upgrading industrial structures, and promoting economic growth in China. However, rapid urbanization poses challenges to China’s resources and environment [29]. China’s urbanization is based on the high consumption of energy and natural resources, resulting in low efficiency, high cost, and the rapid growth of energy and resource consumption [30].

Additionally, urbanland is expanding faster than urban populations in China. Liu et al. [31] believe that with rapid urbanization, urban land use has changed dramatically in space and scale in China. These changes have greatly affected the natural environment. Driven by the expansion of highways around the city and the construction of “development zones”, the rapid urban expansion of big cities has promoted the transformation of farmland for non-agricultural uses [32]. Qian et al. [33] argued that the sustainable use of land resources is facing a huge challenge with the rapid development of urbanization in China. The Chinese government has put forward a new approach to urbanization, which promotes intensive, circular, rural–urban integration, low-carbon, and sustainable development [34]. In this paper, we study the impact of urbanization on eco-efficiency in China.

### 2.3. The Relationship between Urbanization and Eco-Efficiency

A British scholar, E. Howard (1898), put forward the concept of an “idyllic city”, and pioneered research on the connection between urbanization and the environment. Since then, scholars have extensively researched this issue worldwide. Some studies have focused on the effects of urbanization on a single environment factor, such as air quality [35,36,37], carbon emissions [38,39], energy consumption [40,41], water resources [42], transportation [43], or land use [44,45,46]. Some studies have focused on exploring the impacts of urbanization on the overall eco-environment. Yu et al. [47] analyzed the interactions between urbanization and the eco-environment in the urban agglomeration in the middle reaches of the Yangtze River. Wang et al. [48] analyzed the relationship between urbanization and the ecological environment in the Beijing–Tianjin–Hebei region, and believed that before a turning point, the ecological environment deteriorates with urbanization. After the turning point, with the increase of urbanization, the ecological environment improves. Most scholars found that the relationships between urbanization and environment factors are nonlinear.

Some scholars have analyzed the relationship between urbanization and eco-efficiency. The main research methods used include the Impact = Population x Affluence x Technology (IPAT) model or expanded STIRPAT model, which considers the population, affluence, and technology in regression [49]. Huang et al. [19] believed that urban agglomeration is conducive to improving urban eco-efficiency, and urban agglomerations benefit urban eco-efficiency through decentralized effects and structural optimization effects. Li et al. [50] analyzed the impact of urbanization on China’s provincial energy efficiency, and believe that the overall impact of urbanization on energy efficiency in China is negative. Luo et al. [51] used the IPAT model and concluded that there was an asymmetric U-shaped relationship between the urbanization level and eco-efficiency in China. Bai et al. [21] argued that urbanization and ecological efficiency have a “N” relationship. Zheng et al. [52] believe that urbanization has had a positive effect on eco-efficiency in the eastern part of China, while the effect of urbanization on the central and western regions was mainly negative.

However, when investigating the relationship between urbanization and eco-efficiency, most studies have neglected the effects of spatial correlation. In fact, there are extensive links among the population, economy, and environment of different provinces. The closer the provinces are, the closer the links may be [53]. Zhou et al. [54] argued that the eco-efficiency of the Bohai Sea region has significant spatial spillover effects. Guan and Xu [55] believe that China’s energy eco-efficiency has significant spatial agglomeration characteristics. That is to say, the eco-efficiency of a province is affected not only by local factors, but also by the influencing factors and the eco-efficiency of the neighboring areas. Additionally, since China’s official promotion model is based on economic growth, Chinese provincial officials have enormous administrative power and free disposal rights [56]. Some provincial-level local governments pursue economic growth at the expense of the environment [57]. Huang and Xia [58] believe that provincial officials play an important role in the balance between environmental protection and economic growth. Therefore, in this study, we explore the mechanisms behind the effects of urbanization on eco-efficiency in China, using provincial data and from the perspective of spatial econometrics. Likewise, we describe eco-efficiency in the process of urbanization more comprehensively and objectively. Thus, this study provides new insights into the link between urbanization and eco-efficiency.

## 3. Materials and Methods

### 3.1. Study Area

**Figure 1:**
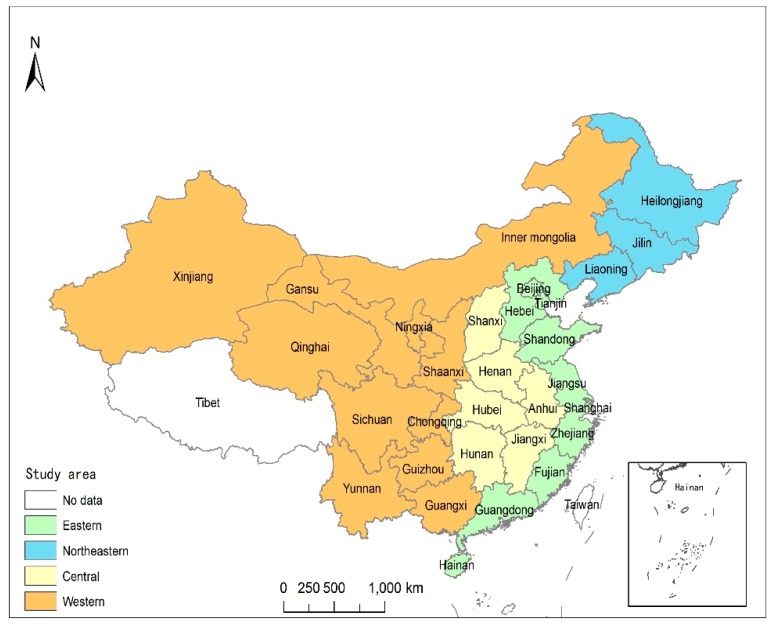
Study area.

China’s urbanization is one of the two most influential events for all mankind in the 21st century [28]. The study area included 30 provinces in China (the sample of this study did not include the Hong Kong, Macao, Taiwan, and Tibet regions due to a lack of data). According to the China Statistic Year Book, the 30 provinces were grouped into four regions: eastern, northeastern, central, and western. Figure 1 shows the study area.

### 3.2. Assessing Eco-Efficiency with the Super-SBM Model

The core concept of eco-efficiency is to develop the regional economy with less input, more output, and without posing an environmental threat [59]. There are many ways to measure eco-efficiency, but the essence is the same for all methods. They all pursue the maximization of desirable outputs, such as per capita GDP and the minimization of undesirable output, such as “the three wastes”, while taking into account the input of production factors, such as labor and capital [24]. The most common method is the DEA method. However, the traditional DEA model does not consider the slack problem of input or output, and cannot achieve a true and effective measurement of the efficiency value when an undesirable output is included. Tone [23] proposed a Super-SBM model, which not only considers the slack improvement, but also solves the problem of the model including an undesirable output. Additionally, the Super-SBM model also allows the effective DMUs to have an efficiency value greater than 1, which solves the difficulty of ranking the DMUs. We drew on the Super-SBM model to calculate eco-efficiency in this paper.

Suppose there are *n* decision-making units putting in *m* factors, then *s*_1_ kinds of desirable output and *s*_2_ kinds of undesirable output will be generated, $x\in R_{m}{,\;y}^{g}\in R_{s1}{,y}^{b}\in R_{s2}$. We defined matrix ${X\;=\;[x}_{1},\cdots {,\;x}_{n\;}]\in R_{m\times n}$, ${\;Y}^{g}{\;=\;[\;y}_{1}^{g},\cdots {,\;y}_{n}^{g}\;]\in R_{s1\times n}$, ${\;Y}^{b}{\;=\;[\;y}_{1}^{b},\cdots {,\;y}_{n}^{b}]\in R_{s2\times n}$, then the set of productive possibilities, excluding DMUs ${(x}_{0}{,\;y}_{0}^{g}{,\;y}_{0}^{b})$, can be expressed as:

(1) $$p\backslash (x_{0},y_{0}^{g},y_{0}^{b})=\left\{(\bar{x},{\bar{y}}^{g},{\bar{y}}^{b})|\bar{x}\geq \sum_{j=1}^{n}\lambda_{j}x_{j},{\bar{y}}^{g}\leq \sum_{j=1}^{n}\lambda_{j}y_{j}^{g},{\bar{y}}^{b}\geq \sum_{j=1}^{n}\lambda_{j}y_{j}^{b},\lambda \geq 0\right\}$$

The Super-SBM model, considering undesirable outputs, is specified as follows:

(2) $$\begin{array}{c} \rho^{*}=\min\frac{\frac{1}{m}\sum_{i=1}^{m}\frac{{\bar{x}}_{i}}{x_{i0}}}{\frac{1}{s_{1}+s_{2}}\left(\sum_{r=1}^{s_{1}}\frac{{\bar{y}}_{r}^{g}}{y_{r0}^{g}}+\sum_{l=1}^{s_{2}}\frac{{\bar{y}}_{u}^{b}}{y_{u0}^{b}}\right)}\\ s.t.\left\{ \begin{array}{l} \bar{x}\geq \sum_{j=1,\neq 0}^{n}\lambda_{j}x_{j},\;j=1,\cdots ,m\\ {\bar{y}}^{g}\leq \sum_{j=1,\neq 0}^{n}\lambda_{j}y_{j}^{g},\;r=1,\cdots ,s_{1}\\ {\bar{y}}^{b}\geq \sum_{j=1,\neq 0}^{n}\lambda_{j}y_{j}^{b},\;u=1,\cdots ,s_{2}\\ \bar{x}\geq x_{0},{\bar{y}}^{g}\leq y_{0}^{g},{\bar{y}}^{b}\geq y_{0}^{b}\\ \lambda \geq 0,\sum_{j=1,\neq 0}^{n}\lambda_{j}=1 \end{array}\right., \end{array}$$

where $\rho^{*}$ is the efficiency value, ${x,\;y}^{g}{,\;y}^{b}$ represent the input vector, the expected output vector, and the non-expected output vector, respectively, and $s^{-}{,\;s}^{g}{,\;s}^{b}\;$ are the slack variables for the input, desirable output, and the undesirable output.

### 3.3. Measuring Spatial Correlation with the Moran Index

The eco-efficiency of a province is affected not only by the internal factors operating in that province alone, but also by factors related to adjacent provinces; that is, there is a spatial correlation between the eco-efficiency of different provinces [60]. We used the Moran index to measure the spatial correlation of eco-efficiency among provinces. The formula of the Moran index is as follows:

(3) $$Moran’sI\;=\;\frac{\sum {}_{i=1}^{n}\sum {}_{j=1}^{n}W_{ij}(Y_{i}-\bar{Y})(Y_{j}-\bar{Y})}{S^{2}\sum {}_{i=1}^{n}\sum {}_{j=1}^{n}W_{ij}},$$

where $\;W_{\mathrm{ij}}\;$ is the spatial weight matrix, $S^{2}\;=\;\frac{1}{n}\overset{n}{\underset{i\;=\;1}{\sum }}(Y_{i}-\bar{Y})$, $\bar{\;Y\;}=\;\frac{1}{n}\overset{n}{\underset{i\;=\;1}{\sum }}\;Y_{i}$, $Y_{i}$, $Y_{j}\;$ are the observed eco-efficiency values of area (province) *i* and area (province) *j*, *n* is the total number of areas (provinces). Moran’s I is between −1 and 1. If the index is >0, it indicates that there is a positive spatial association between variables; that is, high eco-efficiency areas are nearby to each other, and low value areas are adjacent to each other. If the index is <0, it indicates that variables have a negative spatial correlation, and high value areas neighbor low value areas. If Moran’s I = 0, variables are independent of each other and there is no spatial correlation.

### 3.4. Assessing the Effect of Urbanization on Eco-Efficiency with the Spatial Durbin Model

The IPAT model is a classic model for studying the effect of human activities on the ecological environment. The formula is:

(4) I = P × A × T,

where I represents environmental effect, P represents the size of the population, A represents per capita wealth, and T represents the technology. Later, Dietz and Rose extended it to the more general STIRPAT model, which provides a framework for the environmental impact of social and human factors [49]:

(5) $${I\;=\;\alpha P}^{\beta }A^{\delta }T^{\gamma }\mu ,$$

where α  is a constant, β, δ, and γ are the coefficients of the size of the population, per capita wealth, and technology, respectively, and μ  is the random disturbance term.

To reduce the heteroscedasticity of indicators, we transformed the STIRPAT model using a natural logarithm:

(6) lnI = lnα + βlnP + δlnA + γlnT + lnμ.

We extended the STIRPAT model. In this study, *I* was expressed by eco-efficiency, which can be calculated in this paper. According to Luo et al. [51], the impact of the urban population on the environmental carrying capacity is much greater than that of the rural population, so we used the urbanization rate of population (URB) to express *P*. The wealth index *A* was expressed by the per capita gross domestic product (PGDP) of each province. As an indicator of technological level, *T* is not a single variable, but a combination of many other variables that affect the environment (York, 2003) [61]. We defined *T* in three parts: technological innovation (PA), environmental regulation (ER), and openness to the outside world (FDI).

The Moran index, which will be calculated in part four, indicated that the provincial eco-efficiency and urbanization level in China were significantly positively correlated in space, making both parameters suitable for spatial econometric research methods. Since the spatial Durbin model not only takes into account the lag term between independent variables but also the correlation of dependent variables, it is superior to the spatial error model (SEM) and the spatial autoregressive model (SAR) [62,63]. Most of the previous studies have neglected the effects of spatial correlation between urbanization and eco-efficiency and used simple techniques [51]. Therefore, this study takes spatial correlation into account and uses an advanced spatial Durbin to investigate this relationship. The extended formula can be expressed in the following spatial Durbin model:

(7) $$\ln(EE_{it})=\rho \sum_{j=1}^{n}w_{ij}\ln(EE_{it}\;)+\sum_{q=1}^{m}\beta_{iq}\ln(X_{it})+\sum_{j=1}^{n}w_{ij}\ln(X_{it})\theta +u_{i}+\lambda_{t}+\varepsilon_{it},$$

where ${\mathrm{EE}}_{\mathrm{it}}\;$ is the eco-efficiency of China’s different provinces, which will be calculated in part four, $X_{\mathrm{it}}$ is the vectors of the explanatory variables, including the urbanization rate of population and its quadratic terms, the per capita GDP and its quadratic terms, the level of innovation, environmental regulation, and the degree of openness to the outside world, $w_{\mathrm{ij}}{\;\ln(\mathrm{EE}}_{\mathrm{it}})$ is the spatial lag term of eco-efficiency, $w_{\mathrm{ij}}{\;\ln(X}_{\mathrm{it}})\;$ is the spatial lag term of the explanatory variables, and ρ is a spatial autoregressive coefficient, indicating the degree and direction of eco-efficiency of the region affected by the eco-efficiency of neighboring areas. ${\;\beta }_{\mathrm{iq}}$ is the coefficient of the explanatory variables, which reflects the degree and direction of the impact of different explanatory variables on eco-efficiency. θ is the coefficient of the spatial lag term of the explanatory variables, which reflects the degree and direction of the influence of the explanatory variables in neighboring regions on the explanatory variables in the region in question.${\;\mu }_{i}$ is the spatial fixed effect,$\;{\;\lambda }_{t}\;$ is the fixed effect of time,${\;\varepsilon }_{\mathrm{it}}$ is a random disturbance term, and ${\;w}_{\mathrm{ij}}$ is a spatial weight matrix with two forms: (1) spatial adjacency matrix (0–1 matrix): if two places were geographically adjacent, they were assigned a 1, otherwise they were assigned a 0; and (2) inverse geographic distance matrix: referring to the research of Madariaga and Concet [64], the formula of the anti-geographic distance matrix is as follows:

(8) $$w_{ij}=\left\{ \begin{array}{l} 1/d_{ij}\\ 0 \end{array}\right.,$$

where $d_{\mathrm{ij}}$ is the surface distance between province *i* and province *j* calculated by the longitude and latitude of the capital cities of the two provinces.

### 3.5. Input–Output Indicators for Eco-Efficiency, and Data Sources

In the general literature on measuring eco-efficiency, the input indicators of eco-efficiency include three parts: capital, labor, and resources [24,65]. For the capital input, we used the total investment in fixed assets to measure capital according to the research of Huang et al. [66] and Xing et al. [67]. We used Goldsmith’s sustainable inventory method to calculate the fixed assets stock of 30 provinces in China. The depreciation rate was 9.6%, as proposed by Zhang et al. [68]. For the labor input, referring to Zhou et al. [24], we selected the total amount of employed persons to represent labor input. For the resources input, Bai et al. [21] and Zhao et al. [69] used land, water consumption, and electricity consumption to represent resources input. According to their research, we also chose the area of constructed land, the total volume of water consumption, and the total electricity consumption to measure resources input. The output indicators were divided into desirable outputs and undesirable outputs. A desirable output was used to measure the economic benefits. Most literature chooses the gross domestic production (GDP) as the expected output; in this paper, we also used the actual GDP of each province to measure the desirable output, converted to constant prices in 2000. Scholars have chosen different indicators to represent undesirable outputs, but the essence is the same; they mainly try to measure the negative impact on the environment. Zhou et al. [24] used the total volume of waste water, industrial soot emissions, industrial solid waste emissions, and industrial SO_2_ emissions as the variables of the undesirable output indicators. Bai et al. [21] used the waste water discharge, SO_2_ emissions, and soot emissions to measure the negative impact on the environment. Huang et al. [66] and Xing et al. [67] selected different kinds of pollutants to construct the environment index (EI), and used the index as the undesirable output. In this paper, we selected the total amount of produced industrial wastewater, industrial waste gas, and industrial solid waste, which are called the “three industrial wastes”, as the undesirable outputs, following Ma et al. [70] and Yue et al. [71]. Some articles incorporated environmental emissions as input variables into the model [18,21]. Since the Super-SBM model can handle undesirable outputs, we treated the negative impact on the environment as an undesirable output [19]. The details of the input–output indicators are shown in Table 1 below.

Among the above input–output indicators, total amount of investment in fixed assets, total amount of employed persons in urban areas, area of urban build-up, total amount of electricity consumption, total amount of water consumption, and the actual gross domestic product (GDP) indicators were obtained from the *China Statistical Yearbook*. Indicators of industrial wastewater, waste gas, and solid waste were obtained from *China’s Environmental Statistics Yearbook*. Since the disclosure of the industrial three wastes in *China’s Environmental Statistics Yearbook* ended in 2015, the time period of this study was 2005–2015.

### 3.6. Variable Selection for the Spatial Durbin Model, and Data Sources

We selected eco-efficiency, which was calculated in this paper as the explanatory variable of the spatial Durbin model. We also selected the urbanization rate and its quadratic terms as the core explanatory variables. The urbanization rate was expressed by the ratio of the permanent resident population in cities and towns to the total population. The data were obtained from the *Yearbook of China’s Population and Employment Statistics*. We selected four variables as the control variables: Grossman and Krueger [72] found that with the improvement of economic growth, the ecological environment is represented by an inverted U-shape, and proposed the famous Environmental Kuznets Curve. Therefore, we selected per capita GDP and its quadratic items as the first control variable. The per capita GDP was deflated by the GDP deflator based on the level in the year 2000, and its quadratic items were added to verify the Environmental Kuznets Curve. The data were obtained from the statistical yearbooks of each province. Zhou et al. [24] argued that technological innovation has a positive impact on environmental efficiency. We selected innovation level as the second control variable, which was measured by the number of domestic patent applications accepted. The higher the number of patent applications accepted, the stronger the innovation ability. The data originated from the *China Statistical Yearbook*. Porter [73] believed that reasonable environmental regulations can motivate companies to generate “innovative compensation effects”. Lin and Zhu [74] argued that environmental regulation policies have a positive and significant impact on eco-efficiency. Therefore, we selected innovation level as the third control variable, which was expressed as the proportion of investment in industrial pollution control to the total industrial output value in each region. These data were obtained from the *Yearbook of China’s Environmental Statistics*. Barrell and Pain [75] considered that FDI can bring significant technological spillover effects to the host country, while Copeland and Taylor [76] believe that FDI does not bring technological advances, but makes the host countries a “pollution sanctuary” for multinational companies. We selected the degree of openness as the fourth control variable, which was measured as the proportion of foreign direct investment in GDP; this expressed the degree of foreign trade, and tested whether the “pollution paradise” hypothesis exists in China [77]. The data originated from the *China Statistical Yearbook*.

## 4. Results and Discussion

### 4.1. Results of the Eco-Efficiency Calculation

**Figure 2:**
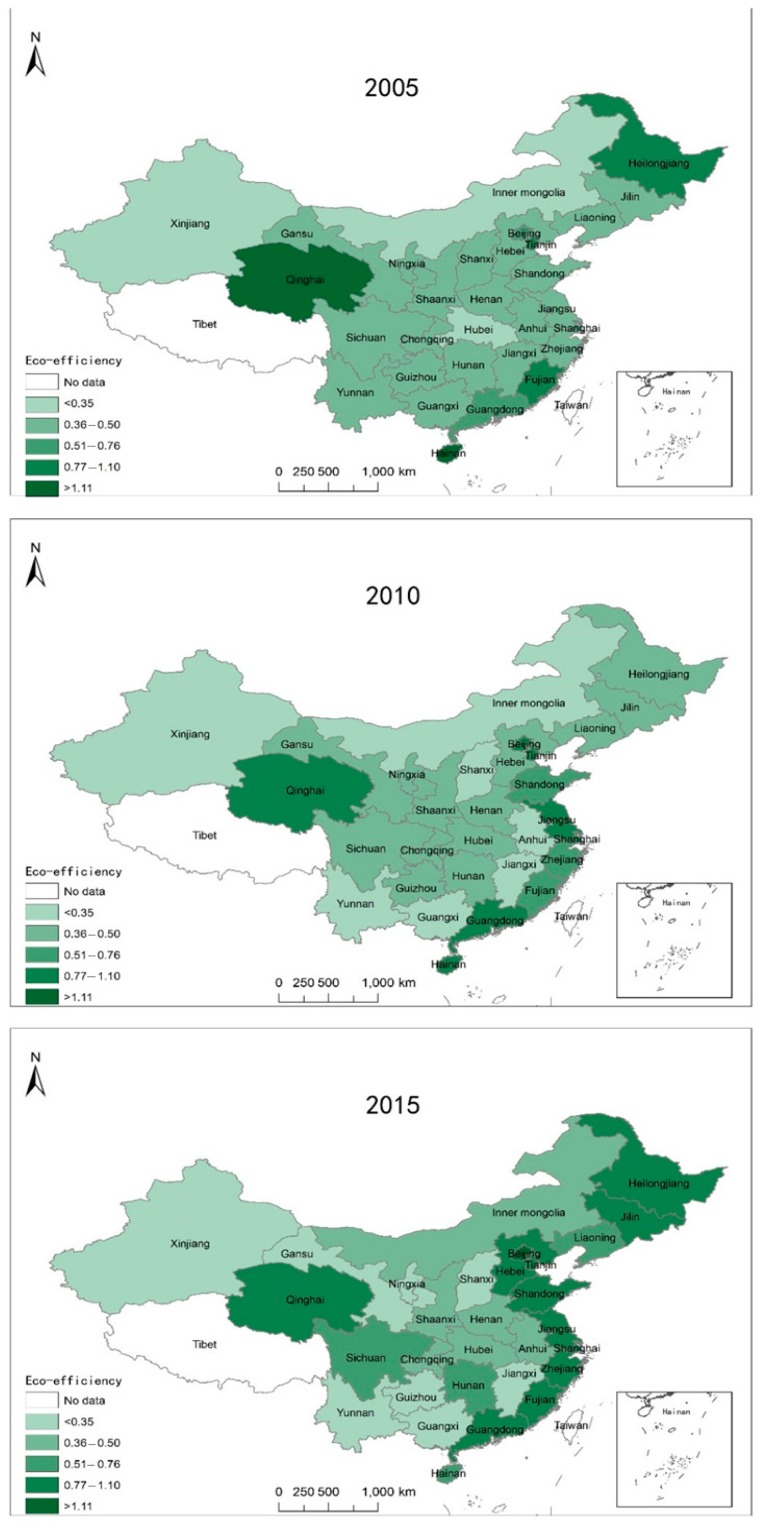
The spatial distribution of eco-efficiency in China in 2005, 2010, and 2015.

We used MaxDEA software (Beijing Realworld Software Company Ltd., Beijing, China) to calculate the eco-efficiency during 2005–2015 based on the Super-SBM model. Since the regional development of China is varied, we analyzed the eco-efficiency from the perspective of the regional distribution of China. The study area included 30 provinces in China (the sample of this study did not include the Hong Kong, Macao, Taiwan, and Tibet regions due to a lack of data). According to the *China Statistical Yearbook*, the 30 provinces could be grouped into four regions: eastern, northeastern, central, and western. The selection of each province was based on data availability, and all provinces were grouped according to the national distribution. Overall, the provincial eco-efficiency in China exhibited a fluctuating and rising trend within the research time, which was consistent with the conclusions of other scholars [21,28]. The eco-efficiency gap among the eastern, northeastern, central, and western regions was obvious, and the gap had a tendency towards further expansion. Figure 2 shows the spatial distribution of the eco-efficiency in 2005, 2010, and 2015 in China.

The eastern region’s eco-efficiency was highest, with an average value of 0.68 or above, and had a rising trend between 2005 and 2015. The north-eastern region’s eco-efficiency was lower than the eastern region, but it was higher than the central and the western regions; the eco-efficiency of the northeastern region rose substantially in 2015. The direction of the trends in eco-efficiency in the central region and western region was not clear. Evidently, there was a large gap between the eastern and western region, and the eco-efficiency level of the western region was slightly higher than that of the central region.

In terms of specific cities and provinces, Beijing, Guangdong, Shanghai, Tianjin, Fujian, Shandong, Hainan, and other eastern developed areas had higher eco-efficiencies, while Ningxia, Gansu, Guizhou, Xinjiang, Inner Mongolia, Shanxi, and other less-developed areas in the western and central regions, and resource-based provinces, had lower eco-efficiency. It is worth mentioning that the eco-efficiency of Qinghai Province, which is in the western region, was relatively high. Except for individual years, the eco-efficiency value was above 1. Although the GDP of Qinghai Province was low, the discharge of pollutants such as waste water and waste gas was also low, placing the area in the high eco-efficiency range, with a low income and low waste discharge. The eco-efficiency of resource-based provinces in central and western China was generally low; this included Gansu, Inner Mongolia, Shanxi, and others. These provinces are dominated by the chemical industry, with high levels of pollution and energy consumption, posing huge challenges to the fragile ecological environment.

### 4.2. Results of Spatial Correlation for Regional Eco-Efficiency and Urbanization

We used the Stata15.0 software (StataCorp LLC, College Station Texas, USA) package to measure the spatial correlation of the provincial eco-efficiency in China during 2005–2015 (Table 2). The results showed that the Moran index of eco-efficiency in all provinces in all years was significantly positive at the 1% level, indicating that the regional eco-efficiency in China has a significant spatial correlation. Thus, the eco-efficiency of a province not only affected the adjacent provinces, but was itself affected by the eco-efficiency of the adjacent provinces. During the research period, the Moran index of urbanization in China was >0 and passed the 1% significance test, indicating that there was also a spatial spillover impact in the level of urbanization in China. Therefore, when establishing econometric models to discuss the impact of urbanization on China’s eco-efficiency, it is necessary to adopt spatial econometric research methods. This study used the spatial Durbin model, based on the results of the Moran index.

### 4.3. Impact of Urbanization on Regional Eco-Efficiency

#### 4.3.1. Results of the Full-Sample Durbin Model

We used the Hausman test to analyze whether the panel data should be a fixed effect model or a random effect model [78]. The results showed that the null hypothesis of stochastic effect could not be rejected, and the spatial Durbin model of stochastic effects was more effective in analyzing the whole sample. To investigate the robustness of the regression results, we used both the spatial adjacency matrix and the anti-geographic distance matrix to measure the regression results. The regression results were very robust (Table 3). Models (1) and (2) used the spatial adjacency matrix for regression; models (3) and (4) used the inverse geographic distance matrix for regression. Compared with models (1) and (3), models (2) and (4) added quadratic terms of urbanization. In this paper, we mainly analyzed the regression results of models (3) and (4).

All coefficients of spatial lag terms were >0, and they were significant at the 1% level, indicating that there was a significant positive spatial correlation among eco-efficiencies of various provinces in China, which suggests that an improvement in eco-efficiency in one province will have a positive effect on that of adjacent provinces. In model (3), the coefficient of urbanization was not significant. After adding the quadratic term for urbanization, the coefficient of urbanization in model (4) changed from insignificant to significant, and the coefficient of the quadratic term was significantly positive at the 1% level. This indicates that the effect of urbanization on eco-efficiency was not a linear relationship, but a U-shaped curve. According to the Environmental Kuznets Curve [72], environmental pollution exhibits an inverted U-shaped curve with economic growth, since the increase in the urbanization rate will promote economic growth, and environmental pollution is negatively correlated with eco-efficiency. We believe that the influence of urbanization on China’s eco-efficiency exhibited a U-curve relationship. Our research conclusions are consistent with Luo et al. [51], who also believe that urbanization and ecological efficiency have a U-curve relationship.

The coefficient of the first term of GDP per capita was negative, and the coefficient of the quadratic term was positive; both were significant at the 1% level. This was consistent with economic phenomena, indicating that the correlation between the economic development and eco-efficiency exhibited a U-curve, verifying the existence of the Environmental Kuznets Curve in China. The coefficient of domestic patent application acceptance (lnpa) was significant at the 5% significance level, but contrary to our expectation, it was negative, indicating that research and development (R&D) innovation did not promote eco-efficiency. The R&D of enterprises may have been directed more toward profit production than toward innovative clean technology [79].

The coefficient of environmental regulation (lner) was positive, but not significant. This indicates that environmental regulation improved eco-efficiency to a certain extent, supporting the Porter hypothesis, which states that environmental regulation can stimulate enterprise investment and environmental technological transformation, and obtain “innovation compensation” [80]. The coefficient of foreign direct investment (lnfdi) was negative, but not significant. This indicates that foreign direct investment had a degree of inhibition of eco-efficiency. The pollution heaven hypothesis was supported to a certain extent, in that foreign developed countries transferred highly-polluting industries to China [81]. Although introducing foreign capital can improve local capital and bring about technological spillover, foreign pollution-intensive industries result in environmental degradation and increase the cost of pollution control in China.

#### 4.3.2. Regional Heterogeneity

Because there were some differences in the development pattern among different provinces, we divided the study area into four regions (eastern, northeastern, central, and western). The specific division scope of the four regions is shown in Figure 1. We investigated the impacts of urbanization on the eco-efficiency of these four regions separately. We used the spatial Durbin model to separately analyze the four regions of China. Model (5) to model (8) were analyzed with the spatial adjacency matrix, and models (9) to (12) with an inverse geographic distance matrix. First, we used the Hausman test to decide whether the fixed or the random effect should be used for this Durbin model. The test results showed that the null hypothesis of the random effect was not supported. Therefore, we adopted the Durbin model of fixed effect for empirical analysis. Table 4 reports the empirical results. The spatial lag coefficients for the eastern, northeastern, and central regions were significantly positive, however, for the western region, they were negative and significant at the 10% significance level. This indicates that there was a significant positive spatial connection among the provincial eco-efficiency levels of the eastern, northeastern, and central regions. In recent years, the development of the western region has been further unbalanced, with high and low values clustering.

There were large regional differences in the estimation results and in the statistical significance of the explanatory variables. Core explanatory variables, such as urbanization level and its quadratic terms (lnurb, (lnurb)^2^), and the estimated coefficients of urbanization level in the eastern, central, and western regions, were negative, and its quadratic terms were positive, indicating a U-curve correlation between urbanization level and eco-efficiency in these three regions; this was consistent with the results for the whole sample. However, urbanization coefficients in northeastern China were the opposite. The first-term coefficient was positive, while the quadratic term coefficient was negative; both were significant at the 5% significance level. This indicates an inverted U-shaped connection between urbanization and eco-efficiency in northeastern China. The reason may be a recent economic recession in the northeastern region, an increase in population outflow, the long-term high proportion of heavy industry, and the relative solidification of the proportion of human capital, all of which have led to an economic relationship characterized by initial stimulation of eco-efficiency with an increase in the urbanization rate, and a later decline along with a continued rise of the urbanization level.

The estimated coefficients of the first control variable—economic development level—and the coefficient of lngrp was significant and negative, and the coefficient of (lngrp)^2^ was significant and positive in the four regions, consistent with the whole sample. The estimation coefficient of the second control variable—level of innovation (lnpa)—was positive in the eastern region, while those of the central, northeastern, and western regions were negative and non-significant. This indicates that innovation has not improved eco-efficiency, except in the eastern region. Estimated coefficients for the third control variable—environmental regulation (lner)—of the eastern and central regions were consistent with the whole sample, and positive, while those of the eastern and western regions were negative, and neither was significant. This indicates that environmental regulation in the eastern and central regions resulted in some improvement in eco-efficiency, while in the northeastern and western regions characteristics of “race to the bottom” were still present [82].

Estimated coefficients of the fourth variable—openness (lnfdi)—for the eastern region were significantly positive at 1%, for central at 5%, and for the northeast and west were negative and significant. This indicates that the eastern and central regions could benefit from opening to the outside world, while the northeastern and western regions have not achieved an improvement of eco-efficiency due to location, economic development level, industrial structure, and other factors. However, with the advancement of the “one belt one road” construction, the two under-developed areas will experience new opportunities.

In the above study, we have already verified the results with the spatial adjacency matrix and the inverse geographic distance matrix. In order to test the robustness of the results, we also used another matrix to refer to highway distance, to retest the regression results at the national level and four regional levels. The matrix was set as the reciprocal of the square of the highway distance between two provincial capital cities, according to Zhang [83]. The results are shown in Table 5. We can see that the results were basically consistent with the above analysis. For the whole country, there was a U-shaped curve relationship between China’s urbanization and eco-efficiency. For the eastern, central, and western regions, the changing trend between urbanization and eco-efficiency was the same as that in the whole country, while there was an inverted U-curve relationship exhibited in the northeastern region. The results of spatial lag coefficients and control variables were also basically consistent with the above analysis. On the whole, the regression results of this paper were robust.

## 5. Conclusions

China’s urbanization is rapidly proceeding and having a significant influence on eco-efficiency. This article investigated the impacts of China’s urbanization on eco-efficiency. We first used the Super-SBM model to measure the eco-efficiency of 30 provinces in China during 2005–2015. Subsequently, we constructed a spatial Durbin model to analyze the effects of urbanization on regional eco-efficiency at the national to regional levels (eastern, northeastern, central, and western). The results showed that the provincial eco-efficiency in China shows a fluctuating and rising trend; the gap between different regions is notable and has an expanding trend. There is an obvious spatial spillover impact on China’s provincial eco-efficiency. The impact of urbanization on China’s eco-efficiency is not a linear relationship, but a U-shaped curve relationship, in which eco-efficiency first falls and then rises. In the early stage of urbanization, labor-intensive industries with low technology levels developed rapidly, resulting in serious environmental pollution and low eco-efficiency. With the improvement in urbanization level, technology improved, pollution control capacity was enhanced, and regional eco-efficiency improved. The change trends the in eastern, central, and western regions were consistent with that in the whole country, while that of the northeastern region was the opposite, exhibiting an inverted U-shaped curve.

The policy implications from the conclusions of this paper are as follows: first, the eco-efficiency in the central region and western region of China are still at a low level, and the gap with the eastern region is widening. The two regions’ local governments need to follow the sustainable development pathway, and change the economic development mode from the traditional extensive development mode of high consumption, high emissions, and high pollution to the development mode of high technology, low resource consumption, low pollution, and low carbon [84]. These two regions’ local governments should also optimize the spatial distribution of urban resource allocation, increase urban green space, and protect the urban environment. Second, owing to the spatial spillover effects of eco-efficiency, we should make the best use of the regional demonstration effect and make this an effective way to improve eco-efficiency. At the same time, local governments, especially neighboring local governments, should be encouraged to cooperate with regional environmental protection, break administrative barriers, realize information sharing, and establish cross-regional ecological compensation mechanisms. Third, provincial governments should change the development mode of urbanization from extensive to intensive development, promote the transformation of low-carbon, green, and sustainable cities, and promote green urbanization development. Finally, all regions should work toward harmonious developments in new urbanization and the ecological environment, according to their current stage of urbanization and local conditions, to improve overall eco-efficiency.

Of course, there were some limitations to this study. The research object of this thesis was limited to the level of the national administrative division. It mainly used provincial panel data, but did not divide the sample area from the urban agglomeration or economic belt. We should see that the impact of urbanization in different economic belts and urban agglomerations on eco-efficiency varies widely. Additionally, urbanization affects the eco-efficiency, and in turn, resource shortages and ecological deterioration also constrain urbanization’s future development. Therefore, in future research, it will be necessary to study the impact of urbanization construction of major economic belts and urban agglomerations on ecological efficiency, according to the specific situation of representative regions in China. At the same time, it is necessary to discuss how to achieve green urbanization under the constraints of protecting the ecological environment.

## Figures and Tables

**Table 1:** Evaluation index system of eco-efficiency.

Layer of Criteria	Layer of Factors	Layer of Indicators	Unit
input	capital	total amount of investment in fixed assets	100 million Yuan
	labor	total amount of employed persons	10 thousand persons
	resources	urban built-up area	km^2^
		total amount of electricity consumption	100 million kWh
		total amount of water consumption	10 thousand tons
desirable output	benefits	GDP	100 million Yuan
undesirable output	negative effect on the environment	total amount of industrial waste water discharged	10 thousand tons
		total amount of industrial waste gas emission	10 thousand tons
		total amount of industrial solid wastes generated	10 thousand tons

**Table 2:** Spatial correlation for regional eco-efficiency and urbanization in China from 2005 to 2015.

Year	Eco-Efficiency			Urbanization Level		
	Moran’s I	*Z*	*p*-Value	Moran’s I	*Z*	*p*-Value
2005	0.074	0.907	0.182	0.363	3.344	0.000
2006	0.343	3.127	0.001	0.364	3.349	0.000
2007	0.329	3.047	0.001	0.371	3.409	0.000
2008	0.353	3.171	0.001	0.375	3.445	0.000
2009	0.344	3.071	0.001	0.387	3.544	0.000
2010	0.360	3.189	0.001	0.384	3.494	0.000
2011	0.391	3.446	0.000	0.375	3.417	0.000
2012	0.285	2.586	0.005	0.367	3.345	0.000
2013	0.305	2.774	0.003	0.369	3.365	0.000
2014	0.263	2.386	0.009	0.369	3.362	0.000
2015	0.381	3.294	0.000	0.384	3.476	0.000

Note: Due to space limitations, only results obtained from the spatial adjacency matrix (i.e., 0–1 matrix) are reported here.

**Table 3:** Estimated results of the Durbin model.

Variable Name	Weight Matrix: Adjacency Matrix		Weight Matrix: Inverse Geographic Distance Matrix	
	Model (1)	Model (2)	Model (3)	Model (4)
lnurb	0.326	−5.078 ***	0.295	−4.545 ***
	(1.10)	(−3.51)	(0.92)	(−3.28)
(lnurb)^2^		0.720 ***		0.650 ***
		(3.80)		(3.55)
lnpgrp	−2.324 ***	−2.179 ***	−2.257 ***	−2.136 ***
	(−6.41)	(−6.51)	(−6.38)	(−6.31)
(lnpgrp)^2^	0.138 ***	0.129 ***	0.135 ***	0.127 ***
	(6.99)	(6.90)	(6.88)	(6.64)
lnpa	−0.084 **	−0.079 **	−0.085 **	−0.085 **
	(−2.00)	(−2.00)	(−2.05)	(−2.19)
lner	0.020	0.020	0.018	0.019
	(1.02)	(1.03)	(0.93)	(1.00)
lnfdi	−0.032	−0.015	−0.037	−0.022
	(−0.95)	(−0.45)	(−1.08)	(−0.67)
_cons	9.255 ***	25.410 ***	8.384 ***	22.880 ***
	(4.63)	(3.32)	(4.72)	(3.88)
Spatial rho	0.370 ***	0.343 ***	0.362 ***	0.332 ***
	(6.40)	(5.94)	(6.23)	(5.64)
Lgt theta	−1.756 ***	−1.440 ***	−1.746 ***	−1.460 ***
	(−5.24)	(−4.82)	(−5.19)	(−4.94)
sigma2_e	0.019 ***	0.019 ***	0.018 ***	0.019 ***
	(3.88)	(3.98)	(4.01)	(4.11)
*N*	330	330	330	330
R^2^	0.3699	0.6306	0.3752	0.6299
LogL	123.82	130.94	126.77	133.36

Note: The figures in brackets are T statistics, and the levels of significance **, and *** are 5%, and 1%, respectively.

**Table 4:** Estimated results of the regional Durbin model.

Variable Name	Eastern	Northeastern	Central	Western	Eastern	Northeastern	Central	Western
	Model (5)	Model (6)	Model (7)	Model (8)	Model (9)	Model (10)	Model (11)	Model (12)
lnurb	−1.581	534.6 **	−8.795	−6.201 ***	−6.056	548.0 **	−5.201	−5.967 **
	(−0.29)	(1.97)	(−1.41)	(−2.82)	(−0.81)	(2.06)	(−1.12)	(−2.44)
(lnurb)^2^	0.240	−65.13 **	0.936	0.834 **	0.725	−66.71 **	0.445	0.809 **
	(0.33)	(−1.97)	(−1.05)	(2.46)	(0.72)	(−2.05)	(−0.64)	(2.17)
lnpgrp	−4.115 ***	−11.21 **	−6.672 ***	−2.108 **	−3.680 ***	−11.33 **	−6.657 ***	−2.065 **
	(−9.51)	(−2.08)	(−4.18)	(−2.27)	(−10.67)	(−2.41)	(−4.22)	(−2.15)
(lnpgrp)^2^	0.164 ***	1.083 **	0.333 ***	0.119 ***	0.155 ***	1.096 **	0.342 ***	0.109 ***
	(4.21)	(2.48)	(5.05)	(3.27)	(4.83)	(2.70)	(4.93)	(3.11)
lnpa	0.103	−0.371	−0.0096	−0.0771	0.176 *	−0.357	−0.0267	−0.0739 *
	(1.05)	(−0.89)	(−0.26)	(−1.63)	(1.87)	(−0.89)	(−0.80)	(−1.66)
lner	0.0321	−0.0525	0.0176	−0.00844	0.0174	−0.0559	0.017	−0.0111
	(0.88)	(−1.06)	(1.03)	(−0.54)	(0.50)	(−1.08)	(0.88)	(−0.80)
lnfdi	0.326 ***	−0.0559	0.0454 **	−0.0296	0.414 ***	−0.0641	0.0412 **	−0.0363
	(4.45)	(−0.71)	(1.97)	(−0.90)	(5.85)	(−0.81)	(2.01)	(−1.08)
Spatial rho	0.139 *	0.331 ***	0.295 ***	−0.158 *	0.176 ***	0.328 ***	0.245 **	−0.122 *
	(1.94)	(2.87)	(2.69)	(−1.72)	(2.77)	(2.69)	(2.31)	(−1.67)
sigma2_e	0.0221 ***	0.0109 *	0.00125 ***	0.00413 **	0.0216 ***	0.0111 *	0.00129 ***	0.00409 **
	(3.22)	(1.92)	(4.10)	(2.27)	(3.23)	(1.94)	(3.75)	(2.53)
*N*	110	33	66	121	110	33	66	121
R^2^	0.5606	0.8390	0.8047	0.642	0.5658	0.8352	0.8097	0.647
LogL	53.03	26.52	125.91	159.99	54.04	26.21	125.18	160.80

Note: The figures in brackets are T statistics, and the levels of significance *, **, and *** are 10%, 5%, and 1%, respectively.

**Table 5:** Robustness test.

Variable Name	The Whole Country	Eastern	Northeast	Central	Western
lnurb	−5.318 ***	−3.932	651.6 ***	−5.982	−6.325 ***
	(−3.35)	(−0.65)	(2.65)	(−1.40)	(−3.79)
(lnurb)^2^	0.771 ***	0.519	−79.84 ***	0.708	0.906 ***
	(3.76)	(0.70)	(−2.66)	(1.14)	(3.70)
lnpgrp	−2.250 ***	−2.921 ***	−16.84 **	−4.193 ***	−1.705 ***
	(−5.28)	(−4.78)	(−2.22)	(−4.80)	(−7.24)
(lnpgrp)^2^	0.134 ***	0.156 ***	1.292 ***	0.242 ***	0.0984 ***
	(5.95)	(4.42)	(2.63)	(4.69)	(6.86)
lnpa	−0.0795 *	0.106	−0.123	−0.0534 **	−0.0418
	(−1.75)	(0.81)	(−0.64)	(−1.98)	(−1.31)
lner	0.0267	0.0196	−0.0536	0.0154	−0.0144
	(1.42)	(0.52)	(−0.83))	(0.90)	(−1.31)
lnfdi	−0.0317	0.273 **	−0.138	0.00320	−0.0372 **
	(−0.97)	(2.14)	(−1.20)	(0.13)	(−2.40)
Spatial rho	0.306 ***	0.227 **	0.0327	0.263 *	−0.0308
	(4.36)	(2.01)	(0.18)	(1.83)	(−0.26)
sigma2_e	0.0199 ***	0.0277 ***	0.00987 ***	0.0015 ***	0.0049 ***
	(4.07)	(6.51)	(4.06)	(5.01)	(7.40)
*N*	330	110	33	66	121
R^2^	0.5686	0.4819	0.8568	0.5161	0.4435
LogL	122.2297	26.8142	29.3659	107.8869	123.3405

Note: The figures in brackets are T statistics, and the levels of significance *, **, and *** are 10%, 5%, and 1%, respectively.
